# Cyclic Thermomechanical Loading of Epoxy Polymer: Modeling with Consideration of Stress Accumulation and Experimental Verification

**DOI:** 10.3390/polym16070910

**Published:** 2024-03-26

**Authors:** Maxim Mishnev, Alexander Korolev, Alexander Zadorin, Vladimir Astashkin

**Affiliations:** Department of Building Construction and Structures, South Ural State University, Chelyabinsk 454080, Russia; zadorinaa@susu.ru (A.Z.); avm1940@mail.ru (V.A.)

**Keywords:** thermal stresses, residual stresses, viscoelasticity, epoxy polymers, structural model

## Abstract

Developing a viscoelastic model for the cyclic thermomechanical loading of thermosetting polymers is the main goal of this study. The model includes memory for residual thermal stresses and can consider stress accumulation across many loading cycles. By considering stress accumulation, we can improve predictions and understand how thermosetting polymers’ stress–strain state changes under cyclic thermomechanical loading. This approach was validated through experimental verification to ensure its applicability in practical engineering scenarios. The experiment showed that the thermosetting polymer can accumulate stress during cycles of heating and mechanical loading during use. The results of the modeling and experiment are compared. The results have led to corrections in the way this model is applied to thermosetting polymers like the epoxy resin in this study. The corrected results matched well with the experimental measurements of stress under cyclic thermomechanical load, with a difference of only 1 to 6%.

## 1. Introduction

Nowadays, there is an expansion of polymer composites’ applications in various industries, including construction. Polymer composites are very useful for making structures like chimneys, gas ducts, tanks, oil pipelines, and more [1,2,3,4,5,6,7,8]. This is possible because of the good combination of strength and weight, the resistance to aggressive substances, and the capability to adjust properties by changing the structure [9,10,11,12,13,14,15].

The inelastic nature of polymeric materials leads to specific effects during cyclic heating and cooling under strain constraints in structures and products made of these materials. The memory of temperature prehistory plays an important role here. It is associated with “freezing” deformation, which occurs when the temperature decreases in a loaded element. As a result, internal residual stresses are formed, which change during service and affect the performance of the structure.

Since the 1970s, there has been a lot of research on the formation of residual stress in thermosetting and thermoplastic polymers and polymer composites. Many studies and publications concern this topic. As usual, it is about residual technological stresses formed during the manufacturing of products.

Residual temperature stresses in polymers and composites are formed due to specific reasons and mechanisms. These are explained in monographs [16,17,18,19,20,21], as well as in review and research publications [22,23,24,25,26]. These factors include temperature variations through structures, different thermal expansions of polymer components, curing process conditions, polymer shrinkage, and interaction with tools.

Residual stress prediction problems can be solved in various ways. In a study from 1976 [22], thermal and moisture residual stresses were analyzed in a layered polymer composite using an elastic approach without considering material viscoelastic properties. However, the majority of other studies on predicting residual stresses in polymers and composites consider the viscoelastic properties, at least of the polymer matrix.

A 1978 paper [27] studied residual thermal stresses in amorphous polymers without reinforcement or filling, caused by their thermomechanical history. It describes the principles of frozen deformations and stresses. Elasticity and viscoelasticity theories are used to predict them, and the effects of macromolecule orientation are taken into account.

In a 1996 paper [28], a procedure for calculating residual thermal stresses during a step-by-step non-isothermal cooling cycle in composite products with a polymer matrix considering its viscoelastic properties was proposed.

To the present day, the topic under consideration has not lost its relevance, as evidenced by many modern works.

For example, in the 2005 paper [29], the finite element method uses the viscoelastic Maxwell model [30] to find the stresses, including residual stresses, in a glass-like material.

In a 2016 paper [31], the problem of calculating the residual stresses in polymer matrix composites appearing during the curing process was solved. For different material states at different stages, the problem was solved in an elastic or viscoelastic formulation.

The 2016 and 2018 papers [32,33] used multiscale finite element modeling to study the viscoelastic–plastic behavior of unidirectional composites with a polymer matrix and to predict the development of residual thermal stresses during curing.

The study of polymers and composites with shape memory is a highly relevant and current topic. This research explores the shape memory effect, which is closely related to relaxation transitions in polymers, viscoelastic thermo-dependent properties, frozen deformations, and the resulting induced stresses. As a result, there are numerous ongoing works in this field, focusing on experimental investigation and the modeling of the viscoelastic behavior of thermosetting and thermoplastic polymers. The paper [34] offers a detailed overview of the latest structural models for polymeric materials. These models simulate viscoelastic processes like creep, relaxation, and the transfer through the glass transition temperature. Within this paper, 19 structural viscoelastic models are described, each with their own specific features and advantages.

The works mentioned above are mainly about mechanical engineering, automotive engineering, aerospace engineering, and medicine. There is not much research on building structures with polymer composites, especially on the issue of thermal stresses during long-term operation at various temperatures. Building structures with polymer composites have unique features that require attention.

The mechanical properties of plastics are influenced by temperature changes, leading to a specific alteration in the stress state of the constrained element. This phenomenon has been observed experimentally [27,35,36] and is characterized by the accumulation of residual stresses, which can be visually represented as a network of cracks in the polymer matrix. Thermal deformations lead to the development of internal stresses. These stresses develop and build up due to the combined effects of creep and relaxation processes. Furthermore, the rate of these processes varies depending on temperature and the level of applied load. Residual stresses can occur during fabrication and continue to change over the lifespan of a structure. Take, for instance, polymer composite structures like chimneys and gas ducts, which may encounter repeated heating and cooling cycles. In these cases, residual stresses can accumulate within individual elements [37,38,39,40].

We propose using an original structural multi-element model to predict how polymers and composites behave under cyclic thermomechanical loading. The model was presented in [36]. The model was tested using a biplastic structure with an inner layer made of unplasticized polyvinyl chloride (PVC), which is a thermoplastic polymer. The prediction of residual thermal stresses in PVC yielded satisfactory results [41].

The present work is mainly aimed at obtaining in the future the results necessary for the design of large-size shell structures (chimneys, liners, etc.) made of polymer composite materials used in gas exhaust ducts of industrial enterprises. The results and methodology obtained, once they are made ready for practical application, including reinforced composites, will enable us to predict with greater accuracy the changes in the stress–strain conditions of the shell structures during their operation. This is particularly important as these structures are subjected to mechanical loads along with cyclically changing temperatures.

To ensure the accurate assessment of the stress–strain state of structures during the design stage, it is crucial to be able to reliably predict residual stress levels throughout the entire operation period. Internal residual stresses play a significant role in both the strength and deformation of polymer composites in large shell structures. The transition to a high-elastic state of thermosetting polymers is strongly influenced by internal stresses and temperature levels [42]. This transition can cause a sudden change in stiffness, impacting the composite’s performance. In highly loaded composite structures experiencing changing temperatures, accumulated residual stresses can cause a sudden decrease in stiffness, leading to the risk of component failure or the loss of stability.

At the current stage, the object of research was an epoxy polymer on anhydride hardener, widely used as a matrix for winding fiberglass structures of chimneys and gas ducts. Unlike PVC, which is a thermoplastic, epoxy resin belongs to thermosetting polymers. Therefore, the application of the proposed model is novel in this case.

In the future, the proposed approach (described in detail below, in the Materials and Methods section) is planned to be extended to reinforced composites with a matrix of thermosetting polymers.

The objectives of this work were as follows:

1. Determine the mechanical parameters of the epoxy polymer being studied for the viscoelastic structural model Kelvin–Voigt.
2. Develop a methodology and conduct experimental studies on the stress state of the investigated polymer under the combined effects of static mechanical load and cyclic changes in temperature.
3. Model the effects of cyclic thermomechanical loading on the epoxy polymer, using the proposed structural model, in conditions that correspond to the experiment.
4. Compare the results of the stress state modeling with the experimental outcomes.

This study brings scientific novelty through two main aspects:

1. It includes experimental data that expose the patterns of epoxy polymer’s stress state formation under cyclic thermomechanical loading.
2. It shows the results from modeling the stress condition of epoxy polymers under repeated thermomechanical loading and compares them with experimental data.

## 2. Materials and Methods

### 2.1. Materials

Experimental studies were carried out on rod samples made of hot-curing epoxy resin on anhydride hardener with the addition of a curing booster.

The following materials were used for manufacturing the experimental specimens:

- KER 828 epoxy resin: epoxy group content (EGC) 5308 mmol/kg, equivalent epoxy weight (EEW) 188.5 g/eq, viscosity at 25 °C 12.7 Pa × s, HCl 116 mg/kg, total chlorine 1011 mg/kg. Manufacturer: KUMHO P&B Chemicals, Seoul, Korea.
- Isomethyltetrahydrophthalic anhydride (IZOMTGFA) (hardener for epoxy resin): viscosity at 25 °C 63 Pa × s, anhydride content 42.4%, volatile fraction content 0.55%, free acid 0.1%. Manufacturer: ASAMBLY Chemicals Company Ltd., Nanjing, China.
- Alkophen (epoxy curing booster): viscosity at 25 °C 150 Pa × s, molecular formula C15H27N3O, molecular weight 265, amine number 600 mg KOH/g. Manufacturer: JSC “Epital”, Moscow, Russian Federation.

One epoxy binder composition was used with the following mass ratio of components:

- Epoxy resin (KER 828)—52.5%.
- Hardener (IZOMTGFA)—44.5%.
- Curing booster (Alkophen)—3%.

**Figure 1:**
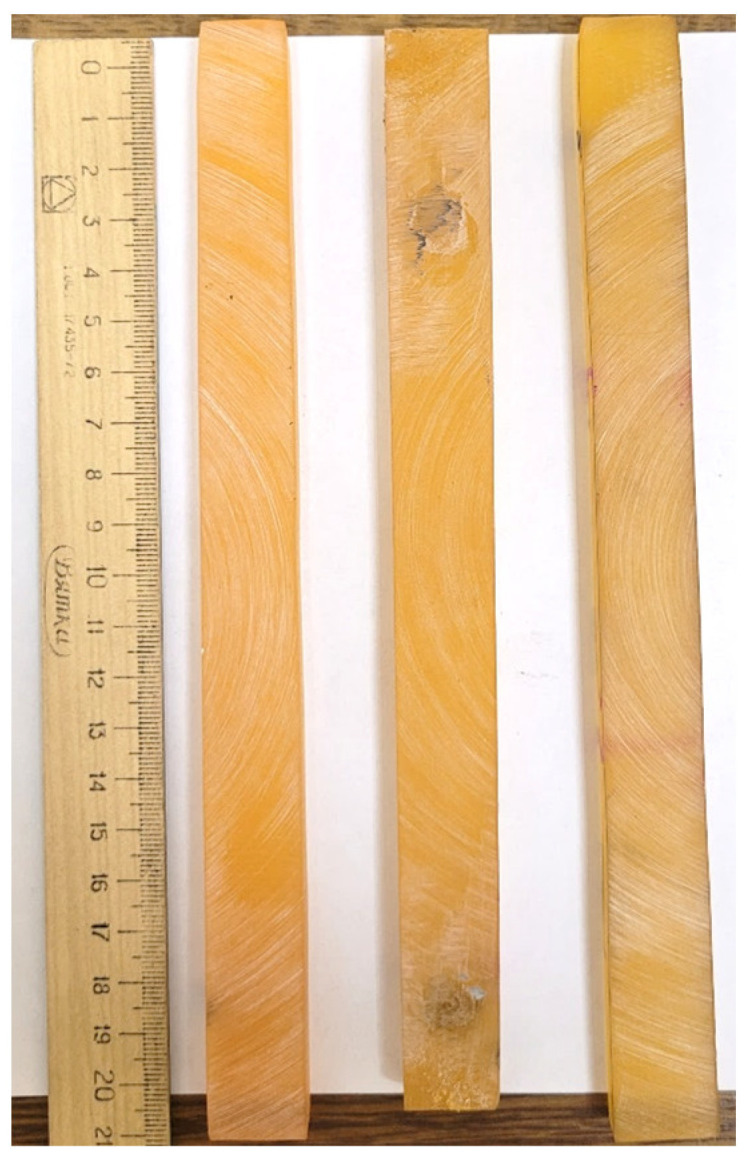
Epoxy rod samples after machining.

We used a Stegler DG-360 mechanical dispersant-homogenizer to mix the binders. It had a 17 mm diameter M-shaped nozzle, which rotated at a speed of 6000 rpm/min. After mixing, the binders were poured into silicone molds and placed in a laboratory oven for curing. The plate samples were precured at 110 °C for 30 min. After initial curing, the rod samples were cut out of the plate and held at 150 °C for 12 h. After curing, the surface of the specimens was further smoothed using an emery wheel. A photo of the specimens is shown in Figure 1.

Three specimens were cut from one plate and tested. See Table 1 for the dimensions of the specimens. The mechanical characteristics of specimen 1 used for modeling verification obtained according to the method described in Section A Description of the Methodology for Determining the Mechanical Characteristics of Samples are given in Table 2.

### 2.2. Methods

#### 2.2.1. Methods of Experimental Research

##### Description of the Experimental Setup

Basic tension tests and cyclic heating and cooling were performed as part of our experimental research for this project. The tests aimed to study how the stress in the specimen changed over time due to thermomechanical loading, creep, and relaxation. Besides the basic tests, it was necessary to experimentally determine the viscoelastic mechanical parameters of the tested specimens for future modeling.

A Tinius Olsen H100KU tensile machine (Horsham, PA, USA) was used to perform tensile tests in combination with thermomechanical loading and to determine the viscoelastic characteristics. These tests were conducted in a specially designed and manufactured thermo-chamber.

**Figure 2:**
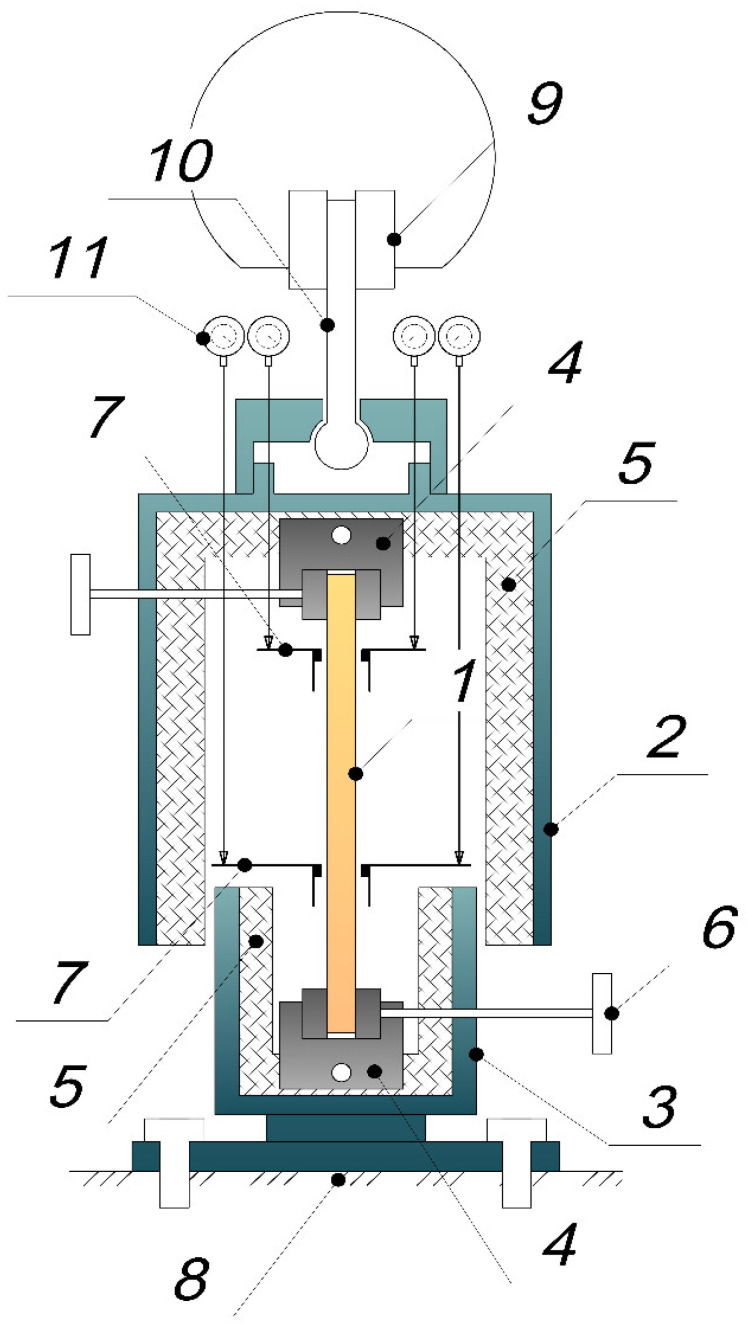
Scheme of the thermal chamber for thermomechanical loading tests.

The Tinius Olsen h100ku machine has a load accuracy of ±0.5% for forces ranging from 0.2% to 100% of the installed force sensor, which has a capacity of 100 kN. The resolution of measuring the crosshead movement was 0.1 mm, with an error of up to 0.01 mm. The scheme of the fabricated thermal chamber is shown in Figure 2. The scheme shows a rod sample 1 clamped in internal clamps 4. The thermal chamber consists of an upper movable part 2 and a lower fixed part 3, rigidly bolted to the fixed base 8 of the Tinius testing machine. The upper part 2 of the thermal chamber is in a suspended state. It is clamped in the upper clamp of the Tinius testing machine by a hinged rod 10. The upper 2 and lower 3 parts are not in contact, which allows for the free deformation of specimen 1 clamped in the inner clamps 4. Crimped angle supports 7 are installed on specimen 1 to hold displacement indicators. This will allow for measuring deformations of the specimen at a fixed length (base) and excluding the influence of slipping in the grips.

The chamber is internally insulated with mineral wool plates and has heating elements and a fan to provide consistent heating when the heaters are on and cooling when they are off. The temperature in the chamber is regulated by a thermostat based on the temperature sensor inside the chamber. During testing, the temperature in the specimen is controlled by an extra thermocouple. It is installed inside a similar epoxy specimen placed next to the specimen being tested.

The thermal chamber explained above is not commercially produced, but it can accurately conduct tensile and compression tests at high temperatures. The accuracy of the specimen load measurement is ensured by the sufficient sensitivity of the Tinius testing machine, because (unlike, for example, industrial DMA analysis instruments) the chamber is designed for testing relatively large-sized specimens. For this reason, less sensitive equipment is required, since the tests are carried out at relatively high loads and are more suitable for the study of building structures with large dimensions.

##### A Description of the Methodology for Determining the Mechanical Characteristics of Samples

**Figure 3:**
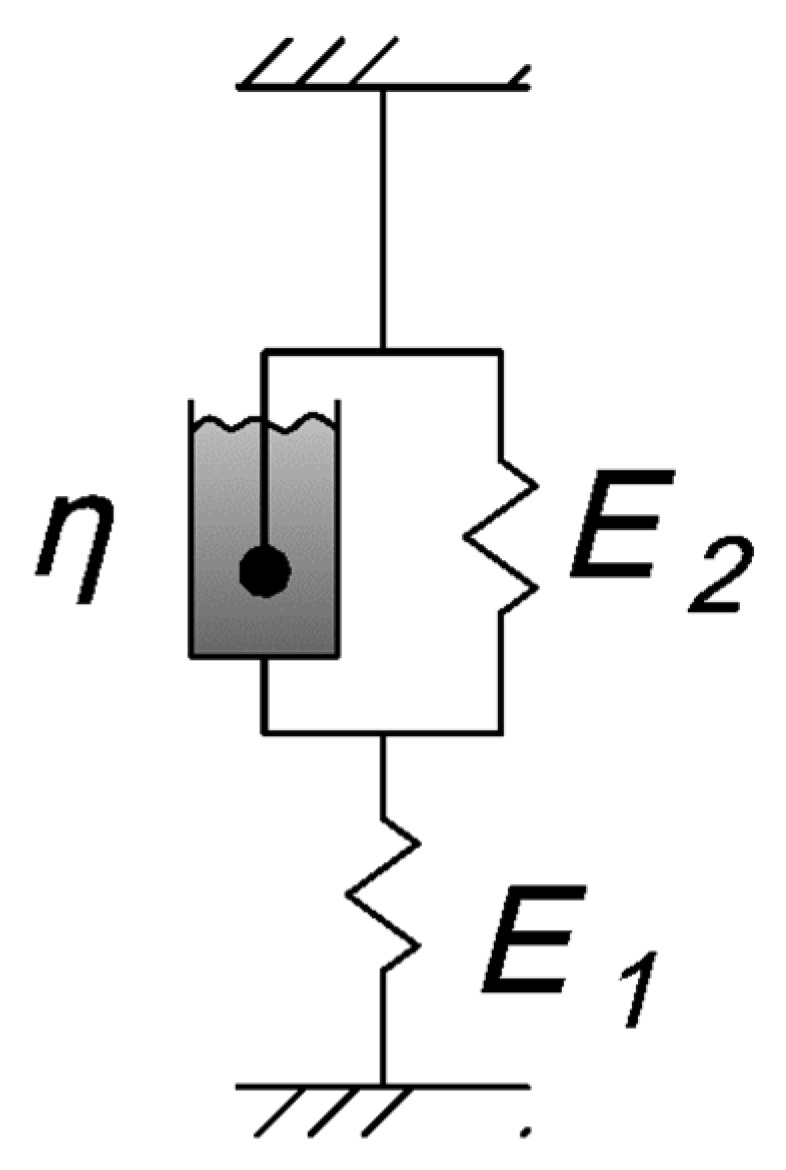
Scheme of the Kelvin–Voigt viscoelastic model (E_1_, E_2_—elastic parameters, η—viscosity coefficient).

The theoretical approach described below and the proposed structural model are based on the use of a three-element viscoelastic material structural model (Figure 3), known in the literature as the Kelvin–Voigt model [30].

The strain law described by this model is as follows:

(1) $$E\cdot n\cdot \dot{\varepsilon }+H\cdot \varepsilon =n\cdot \dot{\sigma }+\sigma ,$$

where *E* = *E*_1_, $H=\frac{E1\cdot E2}{E1+E2}$, and $n=\frac{\eta }{E1+E2}$. The coefficient *E* represents the instantaneous elastic modulus, *H* is the long-term elastic modulus, and *n* is the relaxation time.

**Figure 4:**
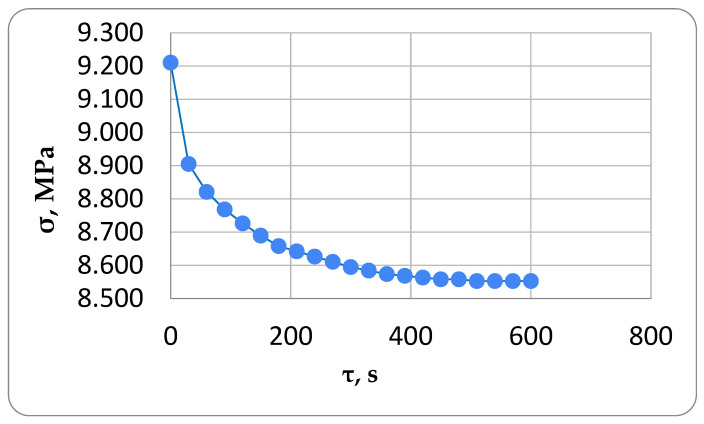
The experimental stress relaxation curve of an epoxy sample for the determination of viscoelastic parameters.

Tensile tests were conducted on the specimens at a consistent temperature of 30 °C to determine the parameters of the Kelvin–Voigt model. The tests were conducted by applying a constant force of 1800 N to the specimen in a heated chamber at 30 °C. Then, the force was gradually reduced due to stress relaxation in the specimen. Indicators recorded the specimen’s displacements on the base during loading to find the parameter *E*_1_ (instantaneous modulus of elasticity). Figure 4 illustrates the stress relaxation in the specimen after applying a tensile force of 1800 N. After the peak load was reached, the force instantly relaxed to 1750 N, which corresponds to a stress of 9.211 MPa based on the specimen’s dimensions of 20 × 9.5 mm. This was considered the initial stress at that moment of time equal to conventional zero.

To determine the mechanical parameters, Equation (1) is transformed to the form as follows [30]:

(2) $$\left.\sigma (\tau )=H\varepsilon_{0}+(\sigma_{0}-H\varepsilon_{0})exp(\frac{-\tau }{m}\right)$$

where *ε*—strains, *σ*—stresses, and *τ*—time.

The problem of determining the mechanical parameters for the Kelvin–Voigt model was solved in the Mathcad package. Arrays of stress values and the time to which these stresses correspond were entered into the package. We initially set the values for the required parameters *E*_2_ and *η* (knowing *E*_1_). Then, we used numerical methods to solve the system of exponential equations and determine the final values for *E*_2_ and *η*.

**Figure 5:**
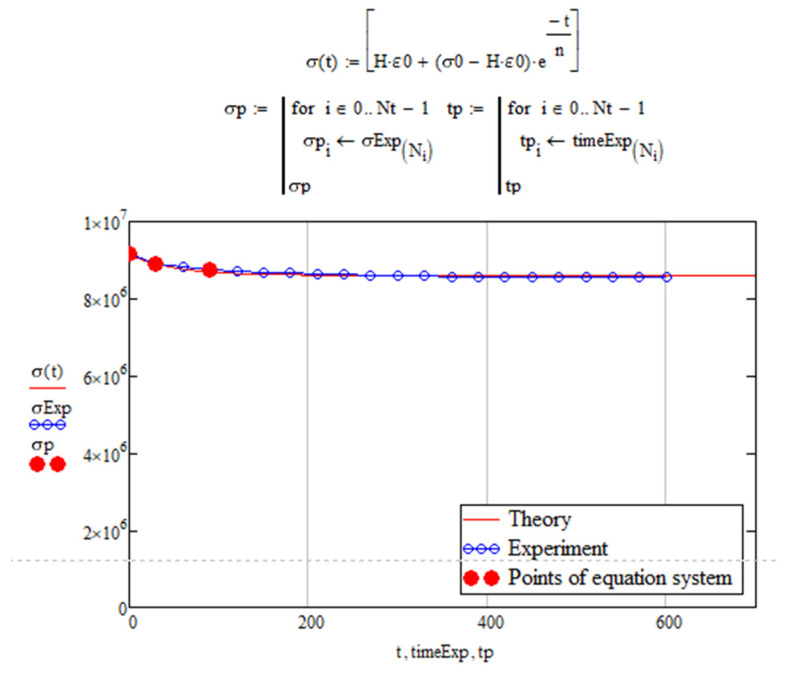
Experimental (in blue) and fitted (in red) relaxation curves.

The numerical method used to solve the problem might not always give values for parameters *E*_2_ and *η* that accurately describe the experimental curve along with the given *E*_1_. To achieve the best visual match between the experimental curve and the curve calculated in Mathcad (Figure 5), we needed to vary the initial values of the parameters and adjust the number of points for the solution. The best parameters for the visual coincidence of the curves were used to model the cyclic thermomechanical loading of the material being studied.

##### A Description of the Methodology of the Experiment on Cyclic Thermomechanical Loading

The experiment consisted of alternately heating and cooling an epoxy rod specimen that was preloaded with a constant tensile load. The stresses in the specimen only changed because of relaxation and thermal expansion/contraction, after the initial loading with tensile load. An initial load of 1800 N was applied to the specimen at a rate of 5 mm/min. The specimen temperature was 30 °C. The load caused an initial elongation of the specimen, which remained constant throughout the experiment. The specimen was heated to 100 °C for 8 min, and then cooled back to 30 °C. The cooling time was about 16 min (+/− 17 s). At each stage of the experiment, we aimed to maintain the same heating and cooling conditions. The force values were recorded on the force meter when the temperatures reached +30 °C and +100 °C. These values were then used to calculate the stresses in the specimen.

**Figure 6:**
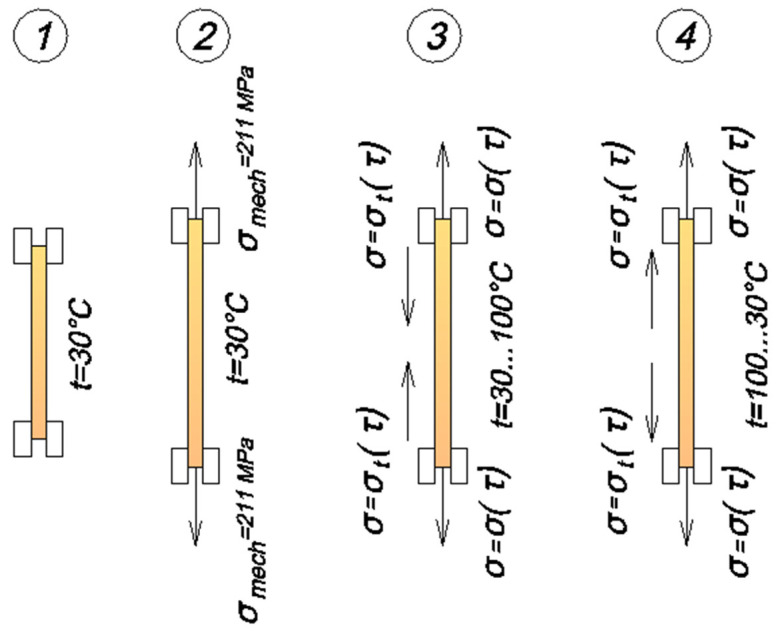
Scheme of experiment steps on cyclic thermomechanical loading.

The cyclic thermomechanical loading experiment consisted of the following steps (Figure 6):

5. The epoxy rod, which had angle crimps already attached, was held in place using the internal clamps from the setup described in Section A Description of the Experimental Setup. The thermal chamber and the specimen installed in it were heated to an initial constant temperature of 30 °C.
6. The specimen was subjected to an initial tensile load of 1800 N (stress 9.211 MPa), and displacements were measured to determine the instantaneous modulus of elasticity E_1_. During the test, the clamp moved at a speed of 5 mm/min. After the load was reached, the clamps stayed in place while the load decreased due to relaxation, and the specimen’s deformation became stable.
7. Once the load of 1800 N was reached, the specimen’s heating mode was activated, raising its temperature to 100 °C in 8 min, achieving a heating rate of 0.1459 °C/s. As the specimen expanded under this heat, the rigid clamping caused compressive thermal stresses to build up, superimposing and reducing the tensile mechanical stresses, as shown by the stress–time curve.
8. When the temperature on the control thermocouple reached 100 °C, the sample cooling mode was activated. The sample was cooled from 100 °C to 30 °C in 16 min (cooling rate was 0.073 °C/s). Upon cooling, the specimen began to shrink, and the compressive temperature stresses that were generated during heating were reduced in the specimen.
9. The specimen went through heating and cooling cycles several times as needed. The force gauge on the testing machine recorded the load values at the peak temperature.

The chosen test scheme involves pre-stretching the specimen using a mechanical load. This keeps the specimen under stress while it is heated, preventing errors caused by backlashes in the testing equipment. Additionally, this scheme allows for evaluating the accuracy of the proposed methodology in a complex stressed state, where both tensile stresses and temperature stresses are relaxing and changing signs.

#### 2.2.2. Methods of Theoretical Research

The structural model of the polymer material consists of multiple elements. It comprises multiple elementary cells connected in parallel, each of which represents the Kelvin–Voigt model mentioned previously. All cells have the same elastic and viscous elements. The main feature that differs this model from other models known to us is that each cell is equipped with a temperature brake that operates at a certain temperature and turns the cell on or off from operation. Cells turn off when the temperature rises and turn on when it falls. Even after turning off, the cells remain active and continue to function through a process called inverse creep. This makes it possible to model the “memory” effect.

The change in the elastic and rheological properties with temperature is explained by the change in the number of working cells with the same mechanical parameters. In the present work, we divide each of the mechanical parameters of the material (*E*_1_, *E*_2_, and *η*) by the number of cells into which the model is divided. Thus, we can only obtain a linear dependence of mechanical properties on temperature, but nonlinearity can be considered if we switch cells not one by one but by several pieces in accordance with the dependence of mechanical characteristic on temperature. In this case, the number of cells should be sufficiently large. There are other possibilities to consider nonlinear dependencies, for example, by adding relevant coefficients, but we do not consider them at this stage. In the proposed formulation, the model can be applied only at temperatures below the glass transition temperature of the material.

Under thermal influence, the model works as follows. The strain **ε**_0_ unrealized in the temperature increment step creates stresses σ in the unit cell, which further relax in time *τ* according to the law [30]:

$$\left.\sigma (\tau )=H\varepsilon_{0}+(\sigma_{0}-H\varepsilon_{0})exp(\frac{-\tau }{m}\right),$$

where *E*_1_, *E*_2_—elastic parameters of the material, *η*—viscosity parameter, *H* = *E*_1_*E*_2_/(*E*_1_ + *E*_2_)—long-term modulus, and *m* = *η*/(*E*_1_ + *E*_2_).

After turning off a cell at time τ*, the stresses are redistributed to other working cells, and the strains in the cells change according to the inverse creep law (we can call this strain as virtual):

(3) $$\left.\varepsilon v(\tau )=(\varepsilon_{0}-\frac{\sigma_{0}}{E_{1}})\cdot exp(-(\tau -\tau *)\cdot \frac{H}{E_{1}m}\right),$$

In this case, the stresses in the turned-off cell become equal to zero, the elastic deformation *ε_e_*, before the moment of brake operation, instantly becomes equal to zero and by absolute value passes into the slip deformation *ε_s_*, and the viscous (rheological) component of the deformation *ε_r_* changes in time, changing *ε_s_*, so that the total deformation at the step, *ε*, equal to the unrealized value of the non-thermal deformation, remains constant.

When the temperature decreases, the cells that were turned off will be turned on again. The virtual strains that were increasing in the turned off state will become stresses. These stresses will be combined with mechanical and thermal stresses and shared evenly among the turned-on cells. In each active cell, the stresses from frozen strains will differ due to their on and off switching happening at different temperatures and times. The accumulated stresses caused by virtual deformations will be the residual stresses formed as a result of cyclic thermomechanical loading.

Thus, if after reaching a certain elevated temperature, the rod is cooled down, the forced-elastic deformations that arose during the heating of the rod are gradually “frozen”. When reheating, the “frozen” forced-elastic deformations gradually unfreeze as the temperature rises, as if partially compensating for the thermal deformations of the rod. The stress curve in this case does not coincide with the stress curve of initial heating.

Real structures can be seen as a system of constrained rods, where the flexibility of the constraint is determined by the design features. Even if the structure is completely homogeneous, the local heating or cooling of its part causes the processes we consider for a constrained rod.

**Figure 7:**
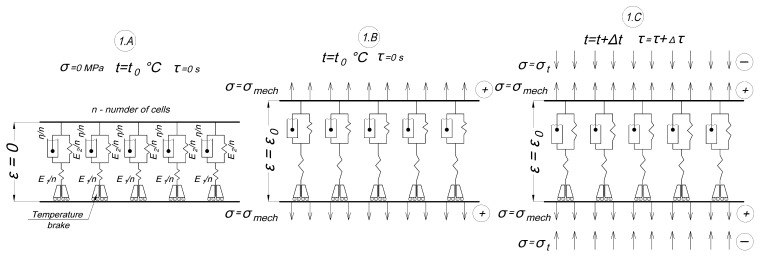
Scheme of the initial stage of the experiment modeled by the proposed structural model.

The scheme below illustrates how we applied the new model in the conditions of our experiment described in Section A Description of the Methodology of the Experiment on Cyclic Thermomechanical Loading. In the scheme, the model is divided into five cells; in real calculations, we divided it into 12 cells. The first stage of the modeling experiment is shown in Figure 7:

- 1.A: initial stage at zero time at initial temperature and zero stresses.
- 1.B: a mechanical tensile load is applied (the time of load increase is not taken into account), mechanical stresses are evenly distributed among all cells, the strain of the specimen has increased to ε_0_, and the temperature is also equal to the initial one.
- 1.C: heating starts, compressive thermal stresses appear, all cells are included, mechanical and thermal stresses are evenly distributed among all cells, and thermal stresses reduce mechanical stresses.

**Figure 8:**
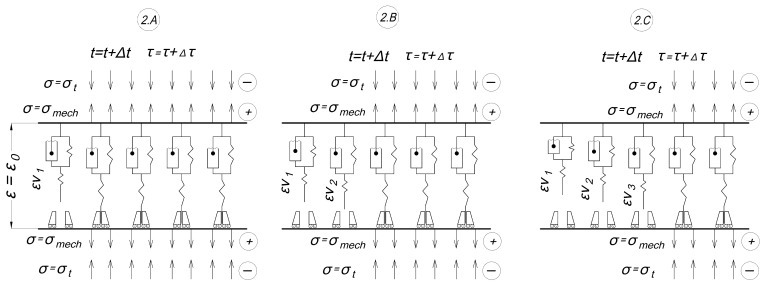
Scheme of the heating stage.

The second stage (heating) of the experiment is shown in Figure 8:

- 2.A: the shutdown temperature of the first cell is reached; it is switched off and deforms according to the inverse creep law (3); before shutdown the cell was stretched, and after shutdown virtual compressive deformations grow in it.
- 2.B, 2.C: the same as in 2.A but with subsequent cells; in the end there are several working cells.

**Figure 9:**
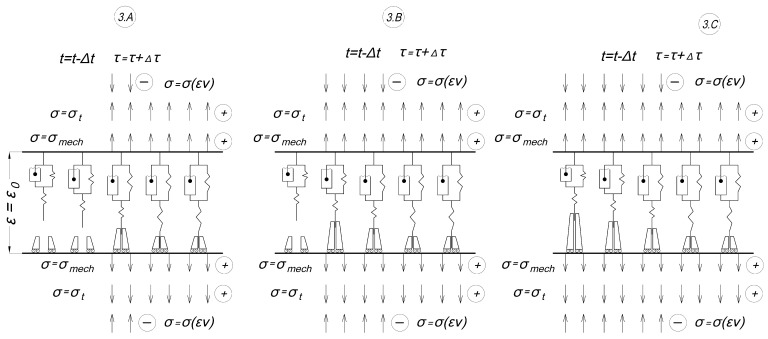
Scheme of the cooling stage.

The third stage (cooling) of the experiment is shown in Figure 9:

- 3.A: cooling is in progress, temperature stresses have changed sign and coincide in the direction with mechanical tensile stresses, the switching temperature of the cell that was turned off last has been reached, and then it is turned on and the strains in it turn into stresses.
- 3.B, 3.C: the same as in 2.A but with subsequent cells; eventually the initial temperature is reached and all cells are turned on, with each having contributed a different value of the residual stresses at turning on.

**Figure 10:**
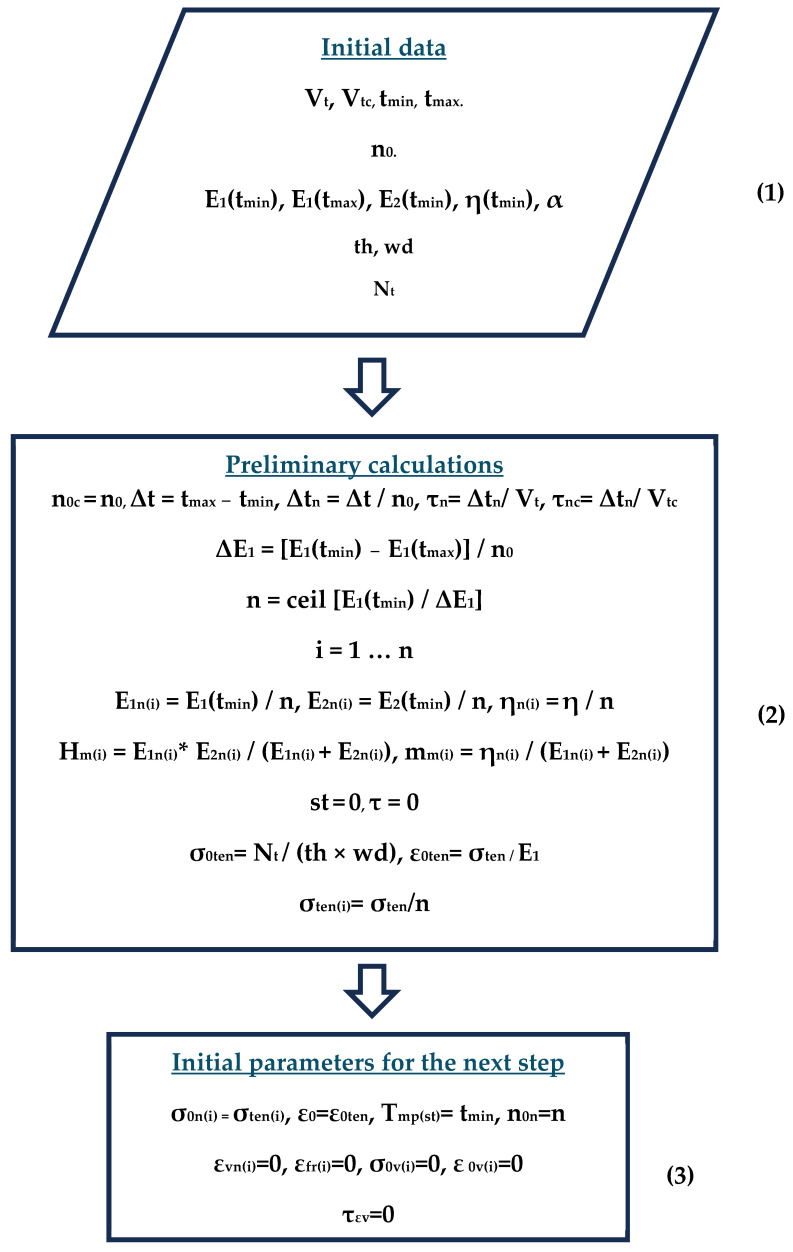
Flowchart of modeling process (beginning).

**Figure 11:**
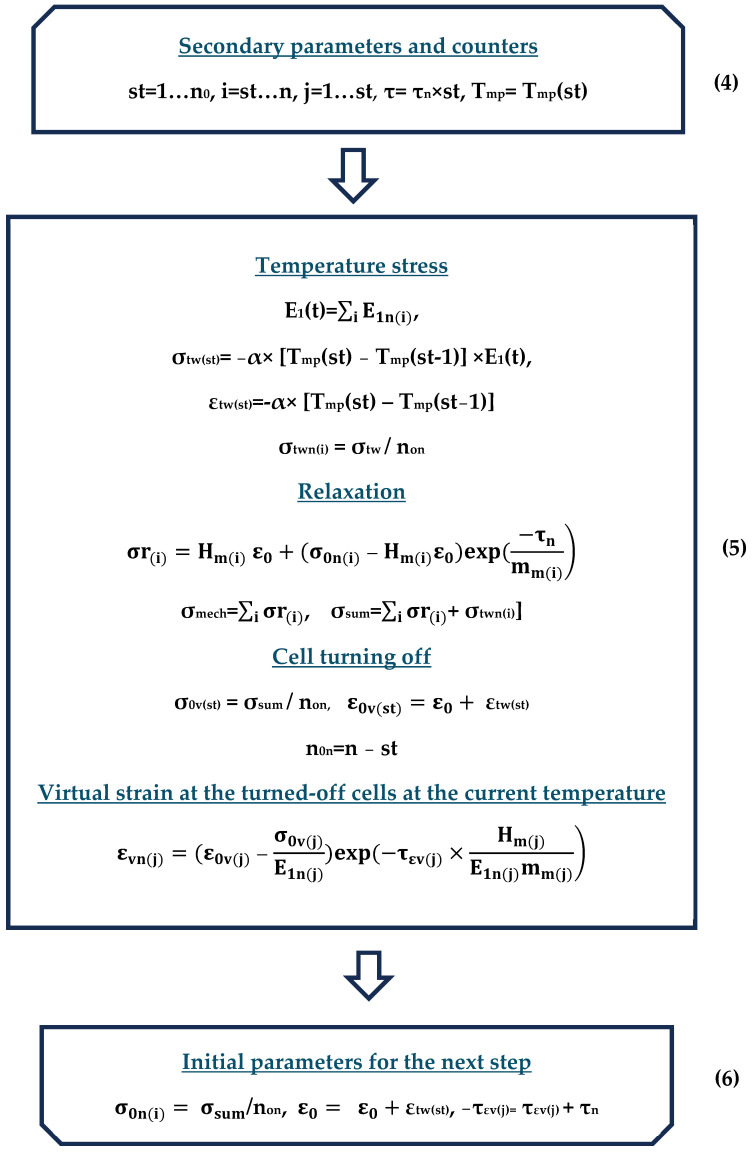
Flowchart of the modeling process (continuation).

**Figure 12:**
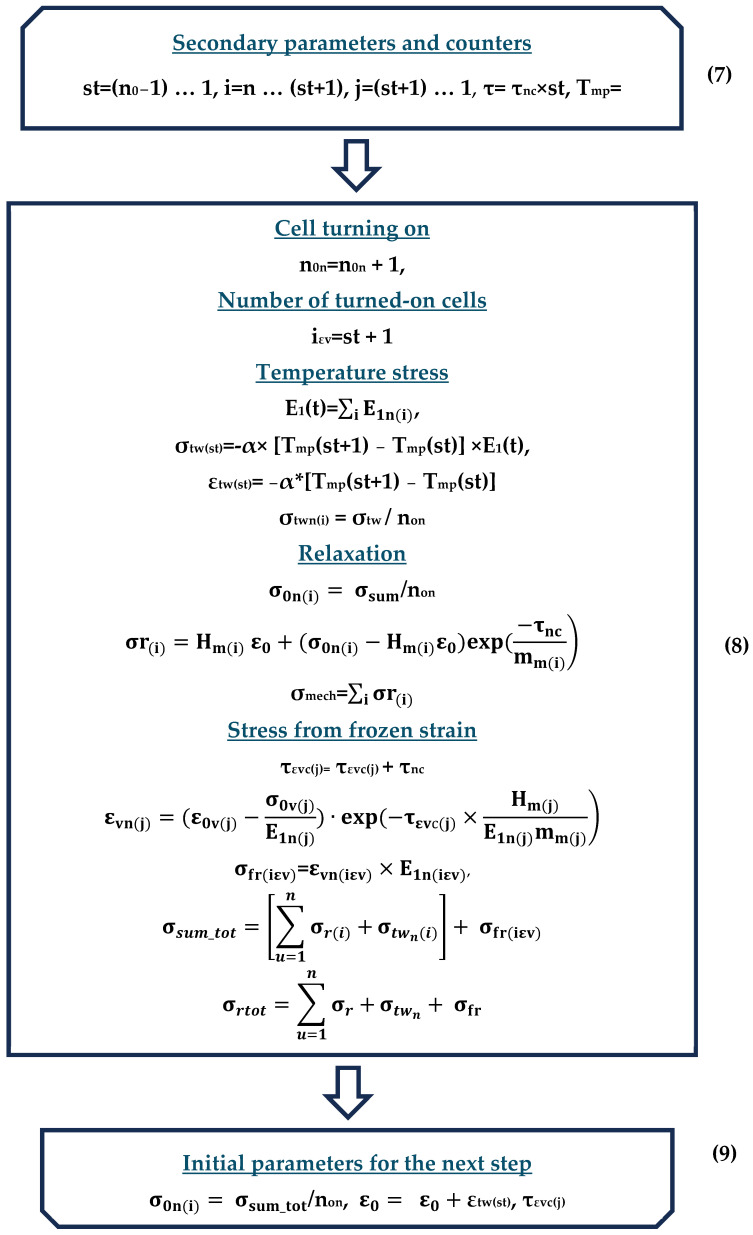
Flowchart of modeling process (finish).

As a result, the experiment described in Section A Description of the Methodology for Determining the Mechanical Characteristics of Samples was modeled as a code in Mathcad, and the calculations are partially presented in Appendix A. The flowchart of the algorithm modeling the experiment is shown in the figures below (Figure 10, Figure 11 and Figure 12); explanations of the flowchart are given in the tables (Table 3, Table 4 and Table 5). The tables provide the numbering and brief descriptions of each block of the flowchart.

## 3. Results

**Figure 13:**
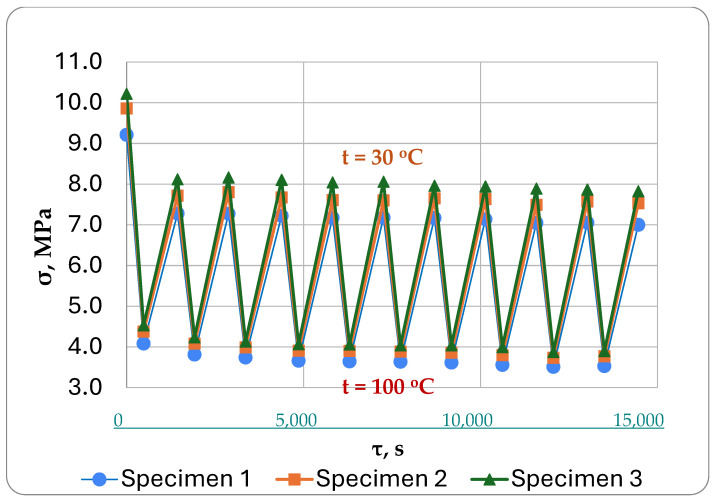
Experimental diagram of stress changes in epoxy samples during heating and cooling cycles under stretch constant mechanical load.

The results of the experimental determination of stresses under cyclic thermomechanical loading for three rod specimens are shown in Figure 13. We can see that they are quite similar both in character and numerical values, which indicates a good repeatability of the results when using this experimental technique.

**Figure 14:**
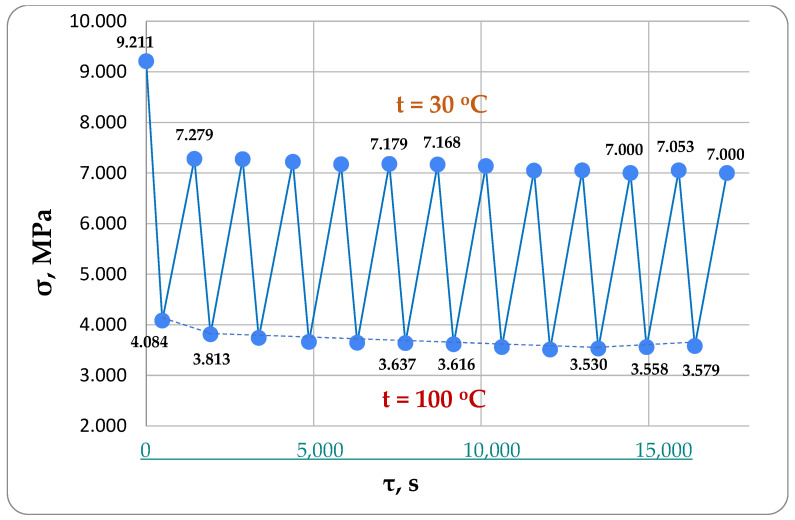
Experimental diagram of stress changes in the specimen №1 used for modeling verification.

Figure 14 shows separately the results for specimen №1, which were directly used to verify the modeling results. These results were obtained by the experimental determination of the mechanical characteristics of the viscoelastic material model using the method described in Section A Description of the Methodology for Determining the Mechanical Characteristics of Samples at 30 °C: E1 = 3100 MPa, E2 = 448,000 MPa, and η = 1.53 * 108 MPa * s. These parameter values were used in further modeling. As a result of the main experiment, graphs of total stress changes in the specimen during the heating and cooling stages for 17,340 s (about 5 h) were obtained. A total of 12 complete heating and cooling cycles were performed, with each heating cycle to 100 °C lasting 8 min and cooling to 30 °C lasting 16 min.

The bottom of Figure 14 shows the stresses when the rod is heated to 100 °C; they represent the difference between the relaxing mechanical tensile stresses and the temperature stresses. After about nine heating cycles, we see a flattening out of the stress values, followed by a slight increase in the modulus (i.e., toward the tensile side), which (presumably) could indicate the residual stress accumulation that we hypothesize may be occurring.

**Figure 15:**
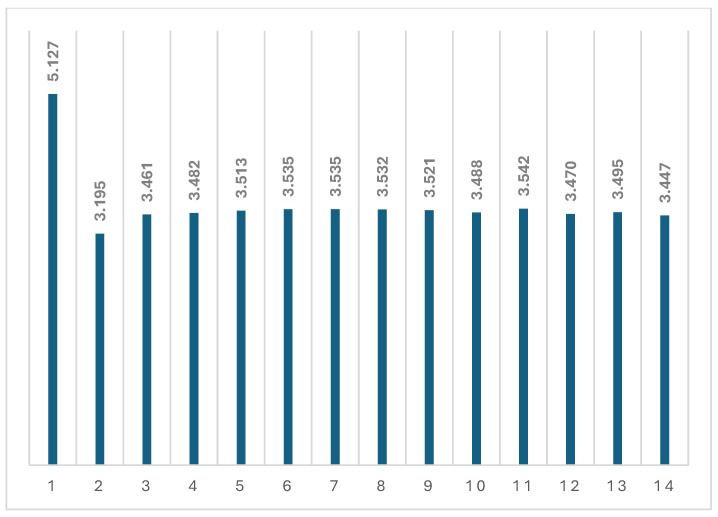
Stress difference (MPa) after each heating and cooling cycle of specimen 1.

In Figure 15, we see a consistent decrease in the difference between the stresses at the lower and upper points of the graph (i.e., at temperatures of 30 °C and 100 °C), which may also indicate the accumulation of residual stresses.

The proposed cyclic thermomechanical loading model was implemented using Mathcad 14.

The following were taken as initial conditions:

- Viscoelastic parameters at 30 °C *E*_1_ = 3100 MPa, *E*_2_ = 448,000 MPa, and *η* = 1.53 * 108 MPa * s.
- The instantaneous modulus of elasticity at 100 °C *E*_1_ = 1200 MPa (taken according to previous works [43,44], where the temperature dependence of the modulus of elasticity of a similar polymer was studied).
- The temperature step was taken as 10 °C.
- The average coefficient of thermal expansion (CTE) was assumed to be 29.5 * 10^−6^ K^−1^ without considering its nonlinear relationship with temperature, based on previous research [45].

The number of cells in the model was determined by calculating the difference in the elastic modulus between 30 °C and 100 °C and dividing it by the chosen temperature step. In this case, the number of cells was 12. Out of the 12 cells, seven turned off one after the other as the temperature rose from 30 °C to 100 °C, while five stayed on and established the stiffness at 100 °C. Assuming a linear dependence of the elastic modulus on temperature [43,44], this approach did not cause significant errors until the glass transition temperature of a polymer was reached.

**Figure A1:**
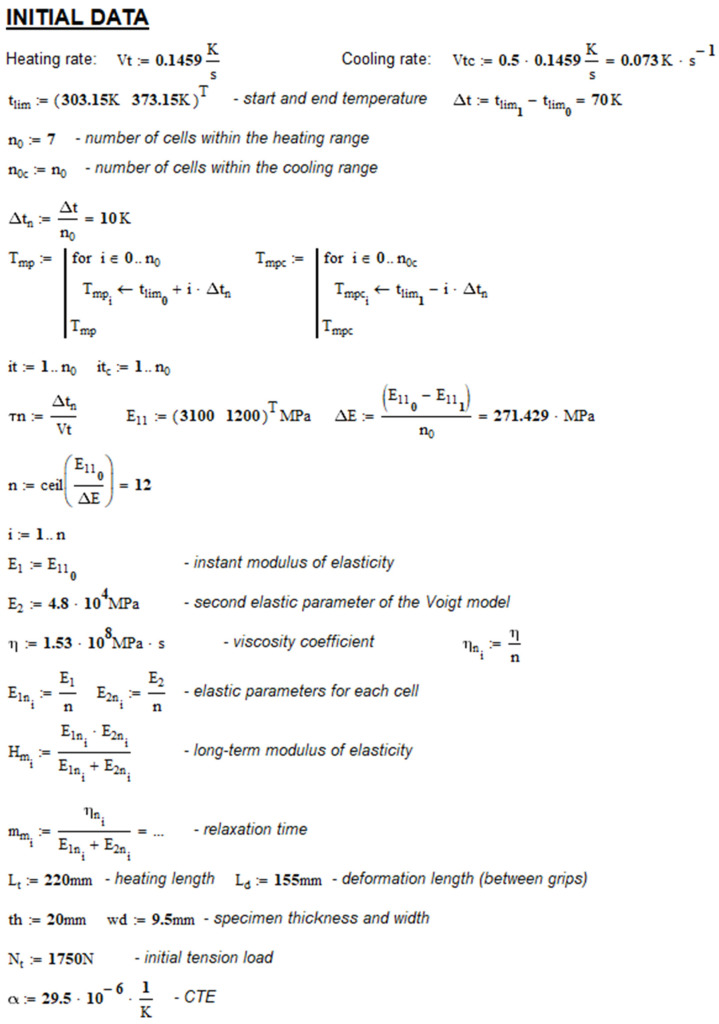
Calculation of initial parameters of structural model.

**Figure A2:**
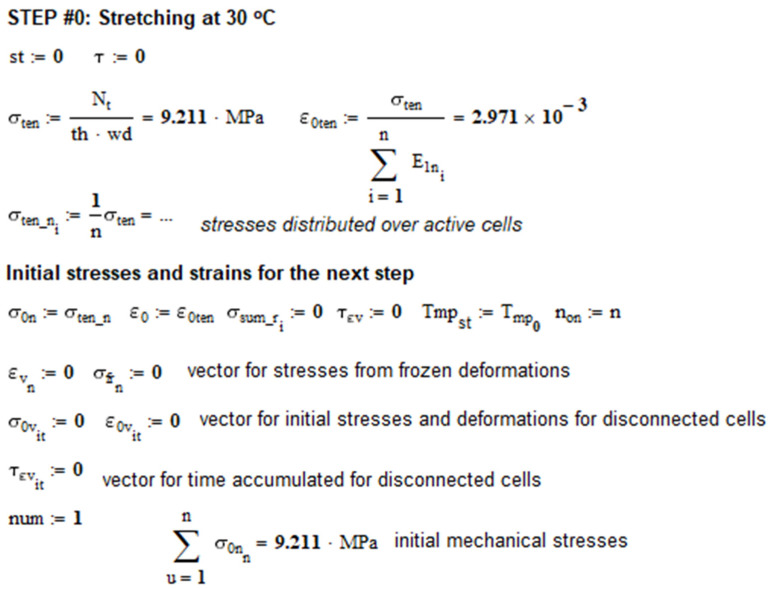
Modeling of initial tensile loading at initial temperature.

**Figure A3:**
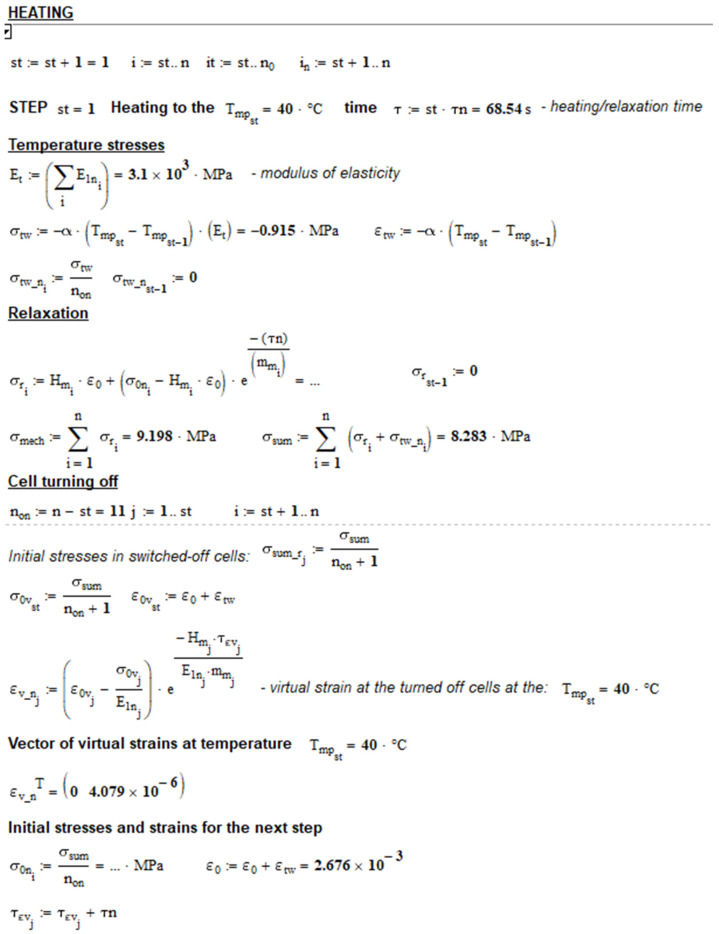
Modeling of heating stage from 30 °C to 40 °C (first cycle).

**Figure A4:**
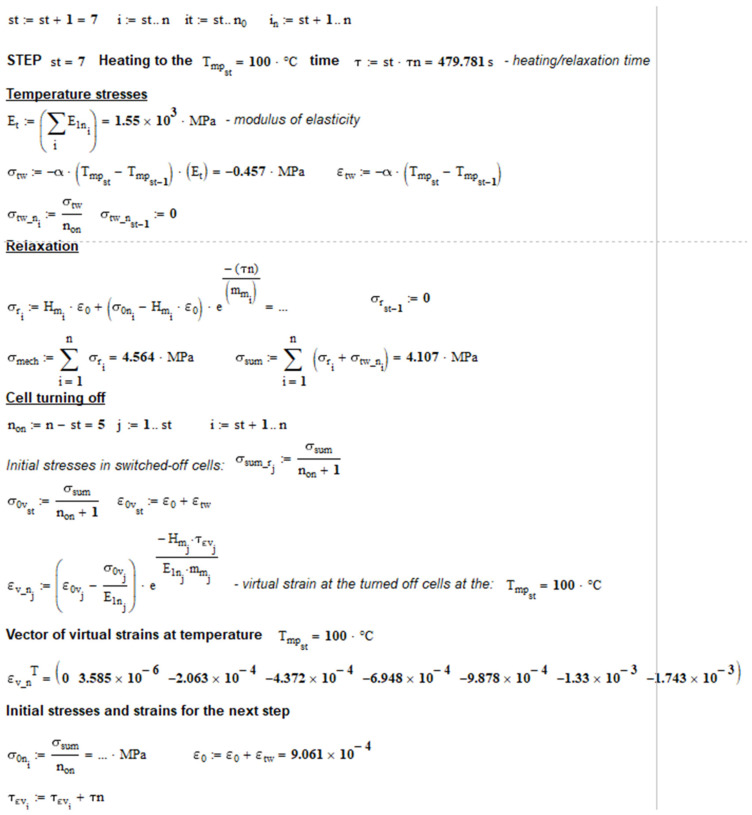
Modeling of the heating stage from 90 °C to 100 °C (first cycle).

**Figure A5:**
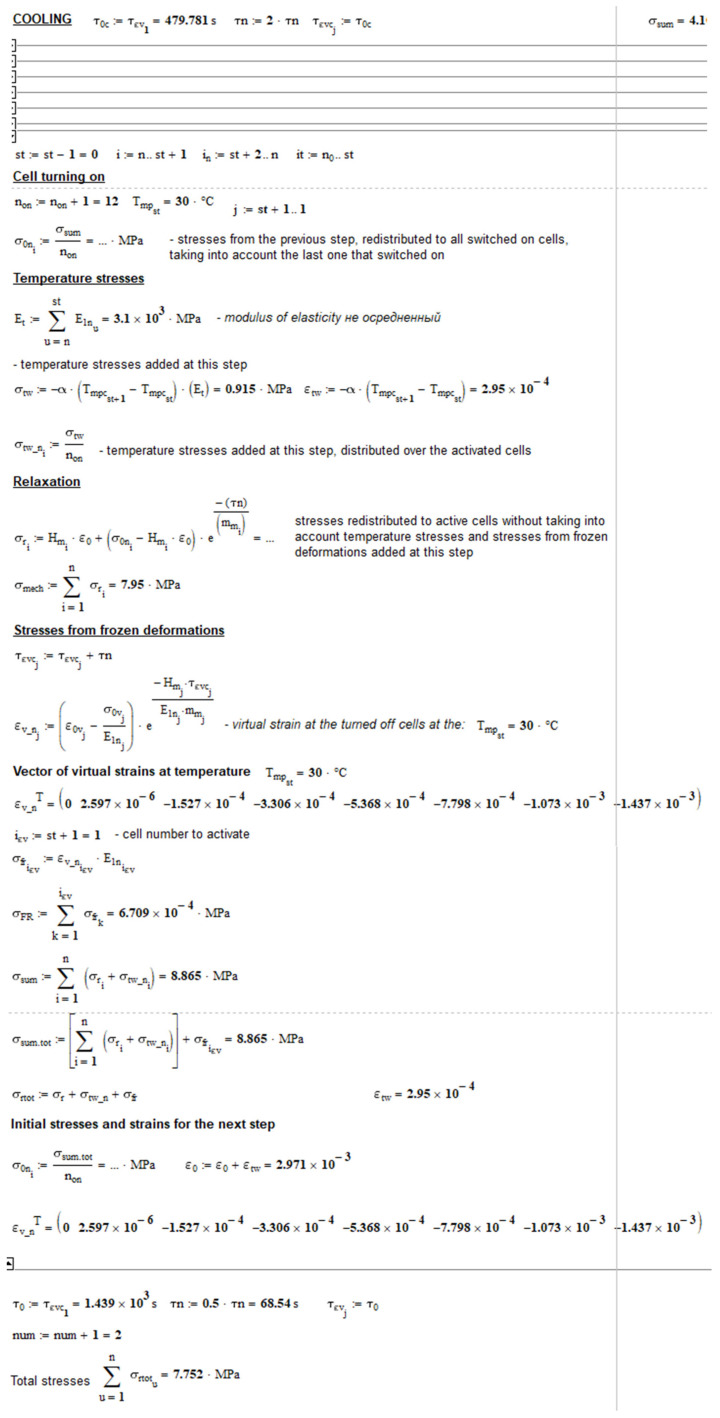
Modeling of the cooling stage from 40 °C to 30 °C (first cycle).

The basic code extractions in Mathcad are shown in Appendix A in Figure A1, Figure A2, Figure A3, Figure A4 and Figure A5.

For comparison purposes, the calculations were performed in several different formulations under the following assumptions:

1. Without considering the stresses from the cells to be turned-off (classical approach).
2. Using the proposed multi-element model, virtual strains in the disconnected cells developed as described above according to the inverse creep law:

$$\left.\varepsilon v(\tau )=(\varepsilon_{0}-\frac{\sigma_{0}}{E_{1}})\cdot exp(-(\tau -\tau *)\cdot \frac{H}{E_{1}m}\right).$$

3. Using the proposed multi-element model, but the virtual deformations in the disconnected cells were developed without considering inverse creep (i.e., fully elastic): $\varepsilon v(\tau )=(\varepsilon_{0}-\frac{\sigma_{0}}{E_{1}})$.

**Figure 16:**
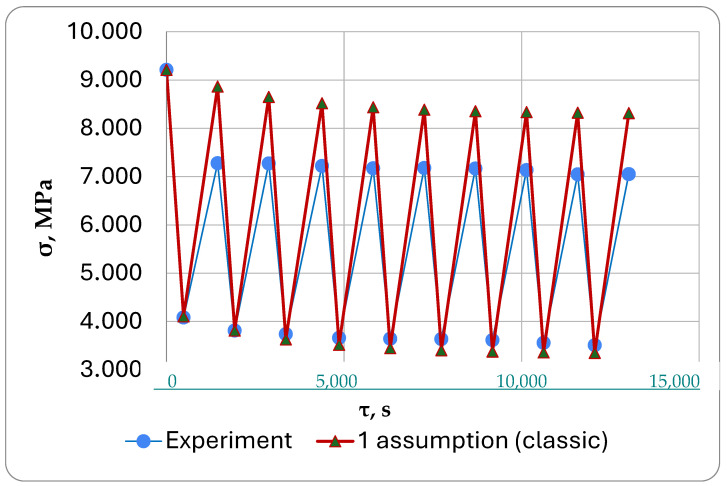
Results of modeling of cyclic thermomechanical loading in first formulation using classical approach.

**Figure 17:**
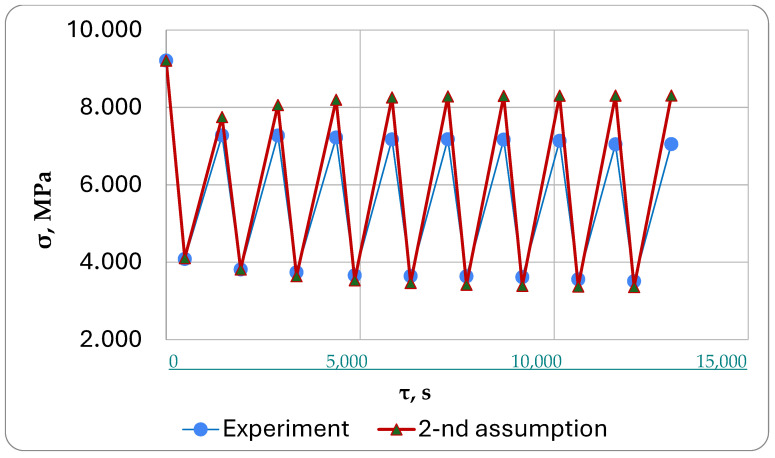
Results of modeling of cyclic thermomechanical loading in second formulation.

**Figure 18:**
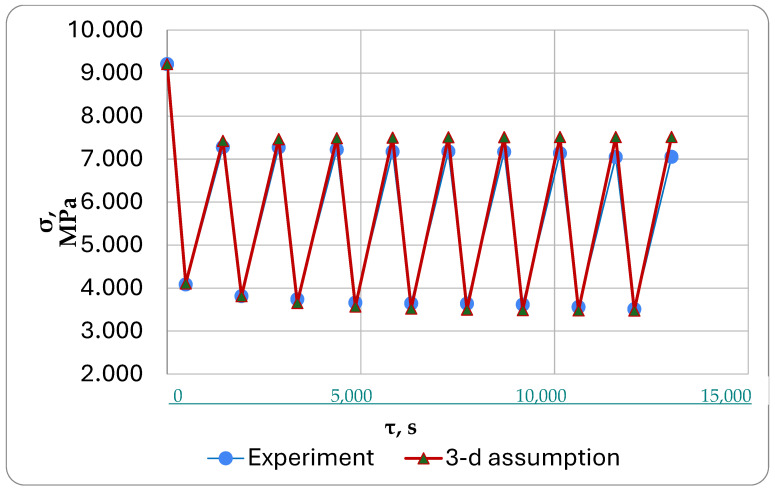
Results of modeling of cyclic thermomechanical loading in third formulation.

The results of the calculations under the first formulation (classical approach) are shown in Figure 16, under the second formulation in Figure 17, and under the third formulation in Figure 18.

The main results of the experiments and modeling were as follows:

- All tested samples showed similar changes in the stress state during cyclic thermomechanical loading.
- The experiment was modeled based on three different assumptions.
- When comparing the modeling results with the experimental data for one of the specimens, it was observed that the simulation under the third assumption provided the closest match (with a stress difference of no more than 6%). Deformations in the switched-off cells of the model were assumed to be purely elastic, instead of following the law of inverse creep as we had first assumed.

## 4. Discussion

The experimental results presented in Section 3 showed the possible existence of residual thermal stresses in thermosetting epoxy polymers after cyclic thermomechanical loading. It should be noted, however, that more experiments are required to verify this. This work was exploratory work, and the main objective was to validate and refine the techniques. When conducting the experiment as described above, it is not possible to directly measure residual stresses. However, it is possible to gather indirect evidence that indicates their presence.

More experiments are needed to study different loading conditions, heating and cooling rates, and the effects of holding time at a constant elevated temperature (which was not conducted in the present research).

We were mainly interested in comparing the simulation results with the experimental results (Figure 16, Figure 17 and Figure 18).

We found a nearly perfect match of stress values from 30 °C to 100 °C (the first descending line in the plots). This indicated that the material’s initial mechanical properties, such as elasticity, viscosity, and the coefficient of thermal expansion, were chosen correctly.

In the first formulation, the theoretical stresses after cooling cycles differed significantly from the experimental ones shown in (Figure 16). After the cooling cycles, the stresses in this formulation were found to be 18…20% lower than the calculated stresses based on the experimental results. This happens because the formulation does not account for the stresses in the turned-off cells caused by reverse creep. In our proposed approach, these stresses are subtracted during cooling, reducing the relaxed mechanical and thermal stresses.

The proposed approach in the second formulation yielded initial heating–cooling cycle results that align closely with the experiment. However, these results diverge as cycles progress and eventually converge with those obtained using the previous classical approach. This happens because there is a significant build-up of internal stresses in this formulation, which continuously increase over time due to inverse creep.

The third formulation (Figure 18) showed the best match with the experiment, with a difference of only 1 to 6%. When the cell is turned off, only reverse elastic deformation happens, which does not increase over time and does not cause significant stress from freezing deformation that reduces the main temperature stresses. One possible explanation for this effect is that when the cells are shut down, they may develop elastic properties like the highly elastic state that the material reaches when it surpasses the glass transition temperature. The deformations in the switch-off cells should develop according to the inverse creep law with specific mechanical parameters (E_1_, E_2_, and η) to be determined for a thermosetting polymer heated above the glass transition temperature instead of in the glassy state. This approach will need to be tested in the future.

## 5. Conclusions

As a result of the work conducted, the methodology of experimentation on cyclic thermomechanical loading has been developed and approved. We conducted experimental studies using specially designed and manufactured nonstandard equipment, which offers a significant advantage over typical alternatives: the accessibility of cost and acceptable accuracy results. A structural multi-element model of the material with the possibility to consider the accumulation of residual stresses has been proposed and tested on the epoxy polymer with anhydride hardener. An approach using this model to predict the formation of the stress–strain state of thermosetting polymers under cyclic thermomechanical loading is proposed.

The obtained theoretical results coincide well enough with the results of the experiment. The directions for further research are outlined, including the development of the proposed approach and its extension for polymeric materials with different compositions and to composites with polymer matrix. It is also reasonable to modify the model so that it can take into account the jump-like transition to a highly elastic state when the glass transition temperature is reached.

## Figures and Tables

**Table 1:** Geometric parameters of samples.

Specimen Number	Section Width, mm	Section Thickness, mm	Length between Clamps, mm
1	20.0	9.5	155
2	19.7	8.5	155
3	19.6	9.0	155

**Table 2:** Mechanical parameters of specimen 1.

Designation	Value	Unit of Measurement	Description
E_1_	3100	MPa	Instantaneous modulus of elasticity at 30 °C
E_1(100)_	1200	MPa	Instantaneous modulus of elasticity at 100 °C [43,44]
E_2_	4.8 × 10^4^	MPa	Elastic parameter of Kelvin–Voigt model at 30 °C
η	1.53 × 10^8^	MPa × s	Viscosity at 30 °C
α	29.5 × 10^−6^	1/K	Coefficient of thermal expansion [45]

**Table 3:** Brief description of block scheme (blocks 1, 2, and 3).

Number Block	Description	Parameters	Parameter Description
**1**	Input of initial data	**V_t_**, **V_tc_**	Heating, cooling rate
		**t_min_**, **t_max_**	Min. and max. heating temperatures
		**n_0_**	Number of cells within the heating range
		**E_1_(t_min_)**, **E_1_(t_max_)**, **E_2_(t_min_)**, **η(t_min_)**, **α**	Instantaneous elastic modulus at minimum and maximum temperature, Kelvin–Voigt model elastic parameter and viscosity at minimum temperature, coefficient of thermal expansion
		**th**, **wd**	Sample thickness and width
		**Nt**	Tensile force on the sample
**2**	Preliminary calculations	**ΔE_1_**	Instantaneous modulus of elasticity corresponding to one cell of the model
		**n**	Total number of cells
		**E_1n(i)_**, **E_2n(i)_**, **η_n(i)_**, **H_m(i)_**, **m_m(i)_**	Mechanical parameters corresponding to the i-th cell of the model (the entry i = 1 …n means that the counter i varies from 1 to n with step 1). As a result, vectors E_1n_, E_2n_, η_n_, H_m_, and m_m_ containing i elements are formed
		**st**, **τ**	Calculation step and its corresponding time
		**σ_0ten_**, **ε_0ten_**	Initial tensile stress and strain
		**σ_ten(i)_**	Initial tensile stress absorbed by one cell
**3**	Initial parameters for the next calculation step	**T_mp(st)_**	Temperature corresponding to step st
		**n_0n_**	Total number of included cells
		**σ_0n(i)_**, **ε_0_**, **ε_vn(i)_**, **ε_fr(i)_**, **σ_0v(i)_**, **ε_0v(i)_**, **τ_εv_**	Initial vectors and parameters for the next calculation step

**Table 4:** Brief description of block scheme (blocks 4, 5, and 6).

Number Block	Description	Parameters	Parameter Description
**4**	Start of the heating cycle at the current step	**St**, **i**, **j**, **τ**, **T_mp_**	Secondary parameters and counters
**5**	Basic calculations in the heating cycle	**E_1_(t)**	Instantaneous modulus of elasticity corresponding to temperature t
		**σ_tw(st)_**, **ε_tw(st)_**	Temperature stresses and strains at step st
		**σ_twn(i)_**	Temperature stresses in the i-th switched-on cell
		**σ_r(i)_**	Stresses in the i-th cell after relaxation for time increment τ_n_
		**σ_0sum_**	Total (mechanical + temperature) stresses summed over all working cells
		**σ_0v(st)_**, **ε_0v(st)_**	Initial stresses and strains for calculating virtual strains at step st
		**n_0n_ = n − st**	Number of enabled cells at step st
		$\varepsilon_{vn(j)}$	Virtual strains in the j-th disconnected cell at the current step
**6**	End of heating cycle at current step	**σ_0n(i)_**, **ε_0_**, **τ_εv(j)_**	Initial parameters for the next calculation step

**Table 5:** Brief description of the block scheme (blocks 7, 8, 9).

Number Block	Description	Parameters	Parameter Description
**7**	Start of the cooling cycle in the current step	**St**, **i**, **j**, **τ**, **T_mp_**	Secondary parameters and counters
**8**	Basic calculations in the cooling cycle	**n_0n_**	Number of enabled cells in the current step
		**E_1_(t)**	Instantaneous modulus of elasticity corresponding to temperature t
		**σ_tw(st)_**, **ε_tw(st)_**	Temperature stresses and deformations at step st
		**σ_twn(i)_**	Temperature stresses in the i-th switched-on cell
		**σ_r(i)_**	Stresses in the i-th cell after relaxation for time increment τ_nc_
		**σ_0mech_**	Stresses summarized over all working cells
		$\varepsilon_{vn(j)}$	Virtual deformations in the j-th disconnected cell at the current step
		$\sigma_{fr(i\varepsilon v)}$	The stresses resulting from virtual deformations in the cell at the moment of its activation after being cooled to the temperature corresponding to the current calculation step
		$\sigma_{sum\_tot}$	Total stresses (including addition of stresses from virtual deformations in the cell keyed at the current step)
		$\sigma_{r\_tot}$	Total stresses in all cells
**9**	End of the cooling cycle in the current step	**σ_0n(i)_**, **ε_0_**, **τ_εvc(j)_**	Initial parameters for the next calculation step

## Data Availability

The authors are ready to provide any data related to the results of this work upon request.
